# Chondromyxoid fibroma management: a single institution experience of 22 cases

**DOI:** 10.1186/1477-7819-12-283

**Published:** 2014-09-12

**Authors:** Jagmeet S Bhamra, Hesham Al-Khateeb, Baljinder S Dhinsa, Panos D Gikas, Roberto Tirabosco, Robin C Pollock, John A Skinner, William J Aston, Asif Saifuddin, Timothy WR Briggs

**Affiliations:** Bone Tumour Unit, The Royal National Orthopaedic Hospital, Stanmore, Middlesex HA7 4LP UK

**Keywords:** Chondromyxoid fibroma, Curettage, Cementation, Functional outcome, Case series, Management, Protocol

## Abstract

**Background:**

Several different strategies have been reported for the treatment of chondromyxoid fibromas, all with variable outcomes and high recurrence rates.

**Methods:**

We report on 22 consecutive cases of chondromyxoid fibromas treated by intralesional curettage, four of which had adjuvant cementation at our institution between 2003 and 2010. We assessed the functional outcome using the Musculoskeletal Tumour Society (MSTS) scoring system.

**Results:**

Nine males and 16 females with a mean age of 36.5 years (range 11 to 73) and a mean follow-up of 60.7 months were included in the study. Local recurrence occurred in two patients (9%) within the first 2 years following the index procedure. This was treated by re-curettage only of the residual defect. Two postoperative complications occurred: a superficial wound infection in one patient and a transient deep peroneal nerve neurapraxia in the other. The mean postoperative MSTS score was 96.7%.

**Conclusions:**

Intralesional curettage and cementation is as an effective treatment strategy for chondromyxoid fibromas, providing satisfactory functional results with a low recurrence rate. Careful case selection with stringent clinical and radiographic follow-up is recommended.

## Background

Chondromyxoid fibroma (CMF) is a rare, benign bone tumour. It is associated with high local recurrence rates with a small risk (<2%) of malignant transformation [1–3]. It was first described in 1948 [4] and constitutes about 1 to 2% of all bone tumours [3]. The tumour is thought to originate from the physeal cartilage plate, given the similar histological findings to chondroblastoma and the reported common involvement of both the epiphysis and metaphysis [5, 6]. CMF can frequently be misdiagnosed histologically as a malignant lesion, due to the finding of atypical pleomorphic hyperchromatic nuclei; however, mitoses are a rarity [5]. The tibia is the most frequently involved bone with other affected sites including the flat bones, facial bones, and bones of the hand and foot [3, 7, 8].

The mean age at diagnosis is approximately 30 years, but the lesion has a wide age distribution [1–3, 9]. CMF development is more aggressive in younger patients and carries a slightly higher predilection for males than females [6]. The age of diagnosis was proposed as a factor for increased recurrence rates, with the suggestion that the reduced resistance of the paediatric thin cortices and spongiosa contributes to the aggressive behaviour of the lesion [6]. Furthermore, there is an increased recurrence in patients less than 15 years of age, with the tumours demonstrating myxoid tissue dominance [10]. In contrast, however, it has been reported that there is no difference in rate of recurrence and age of diagnosis, nor was there any correlation between histological features and increased tendency to recur [6, 11]. Factors proposed in its aetiology include chromosomal abnormalities and immunological factors [12].

The differential diagnosis of CMF includes chondrosarcoma, chondroblastoma, fibrous dysplasia, non-ossifying fibroma, giant cell tumour, aneurysmal bone cyst and simple bone cysts [3, 13–15]. The potential for misdiagnosis may occur in elderly patients, or in cases in which lesions are found in unexpected sites [16].

Historically, wide excision or *en-bloc* segmental resection was recommended, with a reported recurrence rate of 4% [11]. Curettage alone, without adjunctive treatment such as cement, liquid nitrogen and phenol, is reported to result in high recurrence rates (between 20 and 80%) [4, 10, 11]. However, Schajowicz and Gallardo [11] reported no recurrence with combined curettage and bone-grafting, whilst Gherlinzoni and colleagues [6] demonstrated a rate of recurrence of 7%.

**Figure 1:**
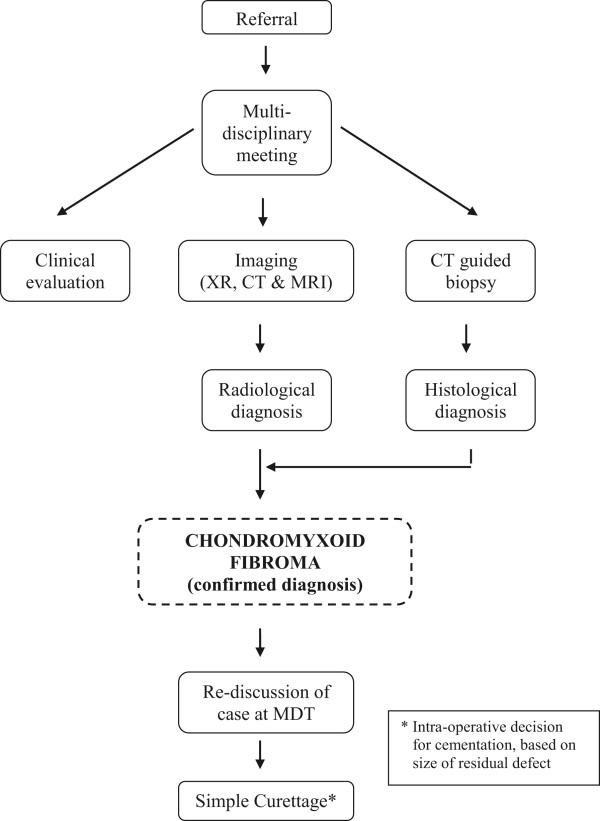
**Algorithm of the standardised approach to treatment of chondromyxoid fibromas at our institution.** CT, computed tomography; MDT, multi-disciplinary team; MRI, magnetic resonance imaging; XR, plain radiographs.

At present, there appears to be no agreed treatment pathway for CMF. This is due to a number of factors, and most likely due to the rarity of this tumour and difficulty in obtaining significant population data. We present a cohort of 22 patients treated in our centre with a standardised treatment approach (Figure 1). Previous studies provide data from a number of treatment modalities, but have relatively small patient numbers and short-term follow-up. In view of the current literature, there is much need for review of the treatment for CMF.

We reviewed our experience of patients with this rare condition and evaluated our outcomes of intralesional curettage and cementation as a treatment strategy.

### Radiology

**Figure 2:**
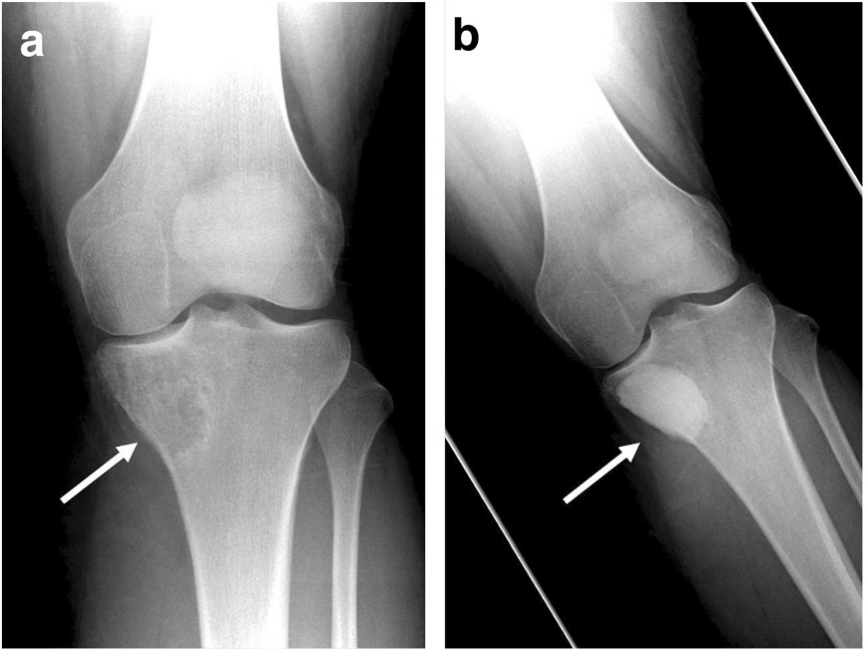
**Radiographs showing CMF of the tibia with postoperative appearances following curettage and cementoma. (a)** Anterior-posterior radiograph of the left knee showing a lytic lesion (arrow) in the medial tibial plateau with a thin, partially sclerotic margin and very mild bone expansion. There is no appreciable matrix mineralisation, and the articular surface of the knee joint is well-preserved. **(b)** Anterior-posterior radiograph of the left knee following curettage and cementation (arrow), showing satisfactory post-operative appearance.

Characteristically, CMF is a sharply defined, eccentric, round or oval geographic (less common) lytic lesion centred in the metaphysis, which measures less than 5 cm at its greatest diameter in most cases. The appearance of CMF in long bones is usually eccentric (Figure 2); however, centrally placed lesions are more common in thin bones (ribs, fibulae, and small tubular bones). The edges are normally scalloped and the endosteal margin is usually well demarcated by a rim of host bone sclerosis (type A margin). Cortical thinning and/or replacement (“expansion”) is quite frequent along with a trabeculated appearance that largely results from ridging of the inner border that surrounds host bone [2, 3, 12, 15, 17, 18].

Pathological fractures rarely occur, with predisposing factors including matrix or intralesional calcification and periosteal new bone production [11, 15]. In our series, we did not encounter any patients presenting with pathological fractures. Current literature suggests that periosteal reaction, in most cases, is as a result of pathologic fractures and is not usually present in CMF [1].

Plain radiographs alone are not sufficient for making a diagnosis of CMF. We routinely perform computed tomography (CT) and magnetic resonance imaging (MRI) in all of our patients. Typical appearances can differ between these radiological modalities with each investigation providing further diagnostic information (Table 1) [3, 19, 20].

**Table 1:** **Typical radiological features of chondromyxoid fibromas as seen on plain radiographs, computed tomography and magnetic resonance imaging**

Radiograph [20]	Computed tomography [15]	Magnetic resonance imaging [19]
Long bones:	In addition to plain film features:	
• Radiolucent	• Calcification may be visible	• Low signal intensity on T1-weighted images
• Lesions may be large	• Septations may be visible	• High signal intensity on T2-weighted images
• May be expansile		
• Lobulated		• Heterogenous enhancement (Gadolinium)
• Geographic lesion with a sclerotic rim		
Small bones (e.g. feet):		
• Osteolytic with “scalloped” bone erosions		
• Bone expansion		
• Coarse trabeculation		

### Histology

**Figure 3:**
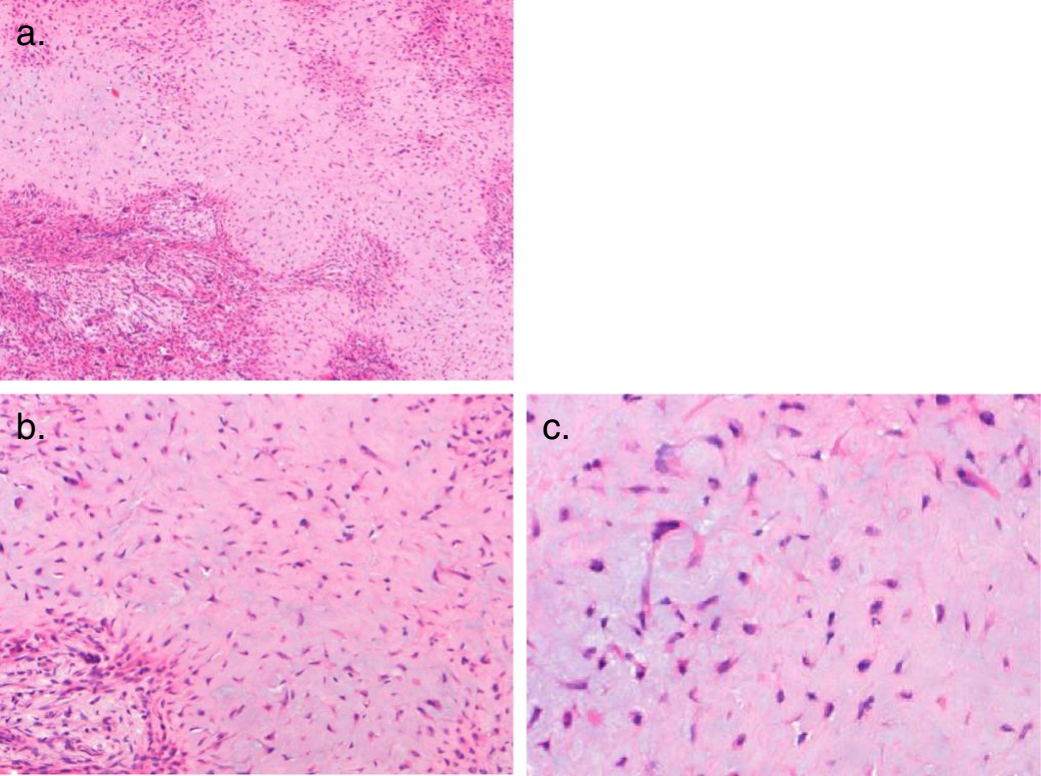
**Histological appearance of typical chondromyxoid fibroma. (a)** Low-power view shows distinct lobulation of the tumour with intervening cellular stromal areas (2× magnification; haematoxylin and eosin stain). **(b)** Medium (10×) and **(c)** high (20×) magnification shows stellate and spindle-shaped cell set in a chondroid matrix (haematoxylin and eosin stain).

The histology of CMF is usually typical, as shown by the Mayo Clinic series [3]. It is a lobulated tumour composed of stellate and spindle cells with variable cellularity, usually more cellular at the periphery of the lobules. The matrix is myxoid and in some cases contains coarse calcification (Figure 3).

The differential diagnosis of CMF includes chondrosarcoma, CMF-like osteosarcomas and chondroblastomas on histology.

## Methods

A search of our regional bone tumour unit database was used to identify patients diagnosed with CMF. Clinical data were collected from the case notes, histology reports, imaging studies, clinic reviews and patient questionnaires. Patients were excluded if the lesion required wide excision or amputation and if they had less than 1 year follow-up. Between 2003 and 2010, 22 consecutive patients were identified with histologically verified CMF who underwent intralesional curettage and cementation of their lesion at our institution. Patients who received bone grafting, shark bite excisions and amputations were excluded from the study due to small numbers.

There were nine males (40.9%) and 13 females (59.1%), with a mean age of 30.6 years (range 11 to 73 years). The mean overall follow-up was 60.7 months (range 8 to 110 months). Ten of the treated lesions occurred in the tibia, four involved the metatarsals, three the calcaneum, three the fibula, one the humerus and one the femur (Table 2).

**Table 2:** **Summary of the site and distribution of chondromyxoid fibromas in our patient cohort**

Site	Cases (n)
Tibia	10
Metatarsal	4
Calcaneum	3
Fibula	3
Humerus	1
Femur	1

Patients were referred to our regional tumour centre and managed by a multidisciplinary team. All patients had persistent pain as the main presenting feature. Interestingly, three patients (13.6%) developed pain after falling. Pre-operative imaging included plain radiographs, CT and MRI. Imaging studies were evaluated by musculoskeletal consultant radiologists for focal bone lesions with geographic bone destruction, a sclerotic rim, lobulated margins, and septation [21].

After planning, patients underwent CT-guided needle biopsy using a Jamsheedi needle and histology specimens were obtained in all cases. The diagnosis of CMF was reached combining radiological, histological (confirmed on needle biopsy) and clinical findings in a multi-disciplinary meeting. The patients included in this study underwent intralesional curettage of their lesion through a cortical window, followed by washout with saline pulsatile lavage. Four out of the 22 patients (18.2%) required adjuvant treatment with bone cement to fill the resultant intraosseous defects. This decision was taken intra-operatively as the size of the residual defect may have resulted in future fractures.

Patients were followed up at regular intervals of 6 months in the first year postoperatively and on an annual basis thereafter for a minimum of 2 years. Plain radiographs were obtained of the operated site at each visit to identify local recurrence. Clinical outcome assessment was performed using the Musculoskeletal Tumour Society (MSTS) scoring system for both upper and lower limbs [21].

The MSTS scoring system is a clinician-completed system assigning numerical values (0–5) for six categories - in the upper limb: pain, function, emotional acceptance, hand positioning, dexterity, and lifting ability; in the lower limb: pain, function, emotional acceptance, ambulatory support, walking ability, and gait. A numerical score and percentage rating was calculated for comparison of our results.

## Results

### Function

The average MSTS score achieved in all 22 patients was 96.7%. The mean upper limb score was 97% in one patient (29/30) whereas the mean lower limb score in 21 patients was 96.3% (range 53 to 100%). All patients were able to perform activities relating to their daily living and occupation. None of the patients had pathological fractures. One patient required long-term analgesia other than that necessary during the postoperative recovery period. This patient had sustained multiple traumatic injuries from a previous road traffic accident and was mobilised pre-operatively with the aid of two crutches. This patient accounted for our lowest MSTS score (15/30).

### Complications

Postoperative complications occurred in only two patients (9.1%), with both cases being classified as early complications. One of the patients developed a superficial wound infection, which resolved with a 7-day course of oral antibiotics, and one patient had a transient deep peroneal nerve neurapraxia with sensory loss after curettage of the first metatarsal which recovered fully.

### Oncological outcome

Local recurrence occurred in three patients (13.6%). Two patients had adequate margins taken at the primary procedure. Recurrence occurred within 6 months in both cases. Treatment was re-curettage in both cases, with no further recurrence to date.

We report one further case of recurrence. In this particular case, recurrence occurred 18 years after primary curettage for CMF. This patient was referred to our unit for further management and re-curettage of the lesion was performed. We were unable to retrieve the initial histology results to ascertain whether inadequate margins were taken at primary surgery.

## Discussion

Current literature data suggest recurrence rates of 3 to 22%. The higher rates are associated with curettage alone; improved results are reported with the use of cementation with or without bone-grafting [4, 5, 8, 11, 16]. We report a recurrence rate of 9.1% in our study. Our results are in keeping with published literature but, more importantly, demonstrate a low recurrence rate with intralesional curettage and cementation. As already described, four out of the 22 cases included in this study received adjuvant cementation. This may have contributed to the low recurrence rate reported in this study, as cement not only provides biomechanical stability but also destroys any residual tumourous cells by thermal injury. We acknowledge that this technique was not standardised in our cohort of patients and is therefore a limitation of the study. Nevertheless, we feel that this was an important step taken intra-operatively to avoid the possible risk of complications. Cementation was to fill the residual intraosseous defect and primarily provides biomechanical stability; however, our experience in treating these tumours suggests that curettage alone is sufficient for the majority of CMF tumours.

The rate of recurrence has been suggested to correlate with age at diagnosis, with younger patients more likely to have recurrences, which may be multiple [4, 5, 8, 11, 16], although reports remain conflicting with regard to this relationship. Due to the relatively low rate of recurrence in our study, it is difficult to verify whether there is in fact an argument to support these claims. Recurrences reported from the two cases in this study demonstrate no sex predilection (50:50 male:female), but do suggest that recurrence occurs in younger patients; with a mean age at recurrence of 19.5 years (range 18 to 23 years). Some cases of recurrence are reported in the literature to occur as late as 19 years from the original diagnosis [3]. We support this statement with a recurrence seen in our patient population at 18 years following primary curettage. One theory postulates that this may be due to the presence of tumorous microlobules deep in bony crevices that can prove difficult to remove by curettage [16]. Malignant transformation is rare, with reported figures suggesting a 1 to 2% risk [16, 21], similar to other benign bone tumours. None of the cases in our series underwent malignant transformation, as confirmed on histological curettage analysis from intra-operative specimens. Of two cases of CMF malignant transformation reported in the recent literature [3], one proceeded to malignant fibrous histiocytoma and the other to fibrosarcoma. The latter had previously undergone radiotherapy. The lack of typical diagnostic features in every case and the propensity for recurrence may lead to misdiagnosis of a malignant tumour. Hence, any lesion labelled as CMF is best confirmed and managed in a specialist bone centre.

Our study demonstrated excellent functional results following intralesional curettage (82%) and cementation (18%) of CMF. The lowest score in our series was secondary to other injuries that the patient sustained, unrelated to the surgery. Our recurrence rate was 9%, which is slightly higher than that reported for curettage and bone-grafting; however, it was better than previous studies that performed curettage alone [8, 11]. In cases where we required cementation in addition to curettage, reconstruction with polymethylmethacrylate bone cement provided immediate stability, avoided the morbidity of autogenous bone graft, and aided the postoperative radiographic evaluation for signs of local recurrence [22].

## Conclusion

In conclusion, CMF is a rare condition with few cases reported in the literature, causing some debate regarding optimal management. Whilst wide excision and *en-bloc* excision procedures report the lowest rate of recurrence, this results in subsequent functional deficit as well as cosmestic concerns. Curettage alone has been reported as showing relatively high rates of recurrence of up to 80%, with an improvement if this is combined with bone-grafting of the defect. This study supports the view that intralesional curettage is an effective treatment strategy for the majority of CMF of long bones with occasional adjuvant cementation being necessary to large intraosseous defects that could result in future recurrence, instability and fracture. We report excellent overall functional results and propose that this treatment option is a safe, minimally invasive procedure that preserves function, has low morbidity, is cost effective, and does not appear to have an adverse effect on long-term outcome. Careful patient selection with stringent clinical and radiographic follow-up is crucial for the successful management of patients receiving curettage with or without cementation as a surgical treatment in a specialist tumour centre.

### Consent

Written informed consent was obtained from the patients for the publication of this report and any accompanying images.

## Abbreviations

CMF: chondromyxoid fibromas
CT: computed tomography
MRI: magnetic resonance imaging
MSTS: Musculoskeletal Tumour Society.
